# Advancing Diagnosis of Liver Cirrhosis: Why Non-invasive Methods Are the Future?

**DOI:** 10.7759/cureus.99071

**Published:** 2025-12-12

**Authors:** Ram Prasad Chaulagain, Khuzin Dinislam, Yelona Shrestha, Dhananjay Kumar Yadav, Abid Ali

**Affiliations:** 1 Internal Medicine, Second Affiliated Hospital of Harbin Medical University, Harbin, CHN; 2 General Chemistry, Bashkir State Medical University, Ufa, RUS; 3 Dermatology, First Affiliated Hospital of Xinxiang Medical University, Xinxiang, CHN; 4 Internal Medicine, Kunming Medical University, Kunming, CHN; 5 Immunology, Harbin Medical University, Harbin, CHN

**Keywords:** artificial intelligence, elastography, liver cirrhosis, multimodal diagnostics, non-invasive diagnosis, serum biomarkers

## Abstract

Liver cirrhosis is the irreversible end-stage of chronic liver disease and remains a significant health burden to the global society. Although traditional methods, such as liver biopsy and hepatic venous pressure gradient (HVPG) measurement, are considered the gold standard, they are limited by bleeding, sampling variability, and patient discomfort. This review explores recent advancements in non-invasive methods (NIMs) for diagnosing and monitoring cirrhosis.

The primary NIMs include serum biomarkers (e.g., Enhanced Liver Fibrosis {ELF} test, Fibrosis-4 {FIB-4} index), which allow for dynamic monitoring of fibrosis progression, and elastography techniques (e.g., vibration-controlled transient elastography {VCTE}, magnetic resonance elastography {MRE}), which demonstrate high accuracy in detecting advanced fibrosis. Further improving diagnostic accuracy and early detection are emerging technologies, such as AI-driven radiomics and multimodal techniques that combine imaging with genetic and epigenetic markers (e.g., circulating cell-free DNA). Despite its benefits - safety, cost-effectiveness, and reproducibility - NIMs have drawbacks, including inconsistent outcomes due to obesity and unequal availability throughout the world. NIMs are increasingly recommended as first-line tools in clinical guidelines, especially in metabolic dysfunction-associated steatotic liver disease (MASLD). To close implementation gaps in the real world, future developments will focus on standardization, point-of-care devices, and the integration of machine learning models. This review emphasizes NIMs as the foundation of a precision hepatology paradigm that is patient-centered. They are poised to replace invasive techniques as standard practice, lowering costs and improving results.

## Introduction and background

Liver cirrhosis, the irreversible end-stage of chronic liver injury, remains a leading cause of global morbidity and mortality, with over two million deaths annually attributed to its complications, including hepatocellular carcinoma (HCC) and portal hypertension [1]. The global burden of cirrhosis has escalated by 13% since 2017, driven by rising rates of metabolic dysfunction-associated steatotic liver disease (MASLD), alcohol-related liver disease, and persistent viral hepatitis, particularly in low- and middle-income countries [2,3]. Notably, MASLD now affects 32% of the global population, mirroring the obesity and diabetes epidemics, and is projected to become the predominant etiology of cirrhosis by 2030 [3,4].

Historically, liver biopsy has been the gold standard for diagnosing cirrhosis, offering histopathological confirmation of fibrosis staging (e.g., meta-analysis of histological data in viral hepatitis {METAVIR F4}). However, its utility is severely limited by invasiveness, sampling variability (10-30% discordance between biopsy passes), and procedural risks, including severe bleeding (0.57%) and mortality (0.009-0.12%) [1,2,4]. Furthermore, biopsies assess <0.002% of the liver parenchyma, leading to misdiagnosis in 20% of cases, particularly in early-stage cirrhosis or heterogeneous fibrosis patterns [1]. These limitations, compounded by patient reluctance and rising healthcare costs, have catalyzed a paradigm shift toward non-invasive methods (NIMs), prioritizing safety, accuracy, and scalability [1,3].

Recent advances in elastography, serum biomarkers, and radiomics have significantly improved the diagnosis of cirrhosis. Vibration-controlled transient elastography (VCTE) and magnetic resonance elastography (MRE) now boast over 90% accuracy (area under the curve (AUC): 0.88-0.96) in identifying advanced fibrosis, surpassing traditional ultrasound and CT, which only achieve sensitivities of 50-70% for detecting late-stage cirrhosis [1,2,4]. When used in conjunction with imaging techniques, serum biomarkers, such as the Enhanced Liver Fibrosis (ELF) score and Fibrosis-4 (FIB-4) index, help reduce diagnostic uncertainty and enable the dynamic tracking of fibrosis progression or regression [3,4]. For example, longitudinal research indicates a 30% decrease in liver stiffness after patients with hepatitis C undergo direct-acting antiviral treatment, which is associated with a reduction in fibrosis [2,4]. Societal guidelines further reinforce the clinical adoption of NIMs. The 2021 European Association for the Study of the Liver (EASL) position statement endorses elastography as a first-line tool for fibrosis staging, while the 2023 American Association for the Study of Liver Diseases (AASLD) guidelines advocate for MRE and VCTE in MASLD management [1,3]. Even with these progressions, obstacles remain. Obesity and liver inflammation complicate elastography outcomes, while differences in global access, especially in areas with limited resources, impede the fair implementation of elastography [1,2]. Emerging technologies, including AI-driven radiomics and microbiome-based diagnostics, offer promising solutions. For example, machine learning models that integrate gut dysbiosis patterns (e.g., reduced Clostridium, increased Prevotella) achieve 81-85% sensitivity for cirrhosis prediction, thereby bridging gaps in early detection [5].

Over the years, the diagnosis of liver cirrhosis has changed considerably. Liver biopsy was first used as the primary diagnostic method for evaluating hepatic disease in the 1950s [6]. In the 1970s, hepatic venous pressure gradient (HVPG) measurement was developed, providing a hemodynamic method for assessing portal hypertension [7]. The application of shear wave theory to medical imaging in 1995 marked a significant technological advancement, establishing the foundation for elastography-based diagnostics [8]. FibroScan (Paris, France: Echosens), a popular non-invasive procedure that uses VCTE, was commercialized in 2001 [9]. Diagnostic precision was significantly improved with the introduction of MRE into clinical practice between 2007 and 2010 [10]. Later, in 2020, radiomics and artificial intelligence were used to diagnose cirrhosis, providing more in-depth, data-driven insights [11]. The most recent development occurred in 2024 when circulating cell-free DNA (cfDNA) analysis and multimodal diagnostic tools that combine imaging and molecular data for a more thorough assessment were adopted [12].

The purpose of this review is to assess how NIMs can improve the diagnosis of cirrhosis. From multiparametric MRI to point-of-care serum testing, we evaluate the efficacy of developing technologies objectively and provide practical guidance on incorporating them into clinical practice. This study supports NIMs as the foundation of a patient-centered, precision hepatology framework by filling in the gaps in standardization and validation.

## Review

Invasive diagnostic approaches

The gold standard for the diagnosis of liver cirrhosis and liver fibrosis has long been considered to be an invasive diagnostic technique. These techniques provide comprehensive histological and hemodynamic data by directly collecting tissue or measuring liver pressures. However, because of the serious hazards associated with their intrusive nature, such as bleeding, infection, and patient discomfort, non-invasive options are becoming more and more popular. Percutaneous liver biopsy, transjugular hepatic venous pressure gradient (HVPG) assessment, percutaneous fine-needle aspiration (FNA), or core biopsy for localized lesions are the most commonly utilized invasive techniques.

Percutaneous Liver Biopsy

For the diagnosis of liver cirrhosis and the stage of fibrosis, liver biopsy is still the gold standard. To directly visualize architectural alterations and fibrosis patterns, a tissue sample from the liver is obtained for histological examination. There are the following three primary methods for doing the procedure: laparoscopic, transjugular, and percutaneous. The most popular technique is percutaneous biopsy, in which a needle is sent through the skin into the liver, usually with the assistance of an ultrasound or CT scan [13]. Patients with coagulopathy or ascites are often candidates for transjugular biopsies, which involve inserting a catheter into the internal jugular vein and then advancing it into the hepatic vein to obtain a tissue sample [14]. Laparoscopic biopsy, performed during laparoscopic surgery, allows for the targeted sampling of specific regions and direct visualization of the liver surface.

Histological analysis of biopsy specimens yields comprehensive details on architectural distortion, fibrosis, inflammation, and steatosis. Upon histological examination, cirrhosis is characterized by the loss of standard lobular architecture, the development of bridging fibrosis, and the formation of regenerating nodules. Liver biopsies have several drawbacks despite their accuracy as diagnostic tools. The surgery carries a risk of complications ranging from 0.1% to 0.5%, including infection and bleeding [15]. Up to 30% of individuals experience pain at the biopsy site, and because of the uneven distribution of fibrosis throughout the liver parenchyma, sampling inaccuracy is a serious problem [16].

Furthermore, the consistency of diagnosis may be impacted by interobserver variation in the interpretation of histological results. Additionally, individuals with significant coagulopathy or hemodynamic instability, recalcitrant patients, and hepatic vascular tumors should not get a liver biopsy [17]. Gastroenterologists and hepatologists formerly handled liver biopsies. However, more and more liver biopsies are being done by radiologists. It is unclear how the shift will impact patient outcomes, safety, and fellow training.

Transjugular Liver Biopsy

An alternative to percutaneous biopsy is transjugular liver biopsy, especially in patients with coagulopathy or ascites, where the latter entails a higher risk of bleeding. A catheter is passed via the right atrium and into the hepatic vein after being placed into the internal jugular vein. Under fluoroscopic guidance, a biopsy needle is inserted via the catheter to collect samples of liver tissue [18]. The HVPG can be measured simultaneously with a transjugular biopsy, which yields important details on the severity of portal hypertension. However, the expense, radiation exposure to the patient, lengthier treatment duration compared to the more popular percutaneous liver biopsy, and requirement for a skilled interventional radiologist are the relative drawbacks of transjugular liver biopsy [19]. There have also been reports of procedural complications such as arrhythmias, internal vascular damage, and hemorrhage.

Hepatic Venous Pressure Gradient (HVPG) Measurement

The venous pressure gradient is an intrusive method that necessitates venous catheterization and is now the gold standard for evaluating HVPG. HVPG typically falls between 1 mmHg and 5 mmHg, and when it hits 10 mmHg or above, it becomes clinically relevant. Inadequate coagulation levels and iodinated contrast allergy are the only related contraindications to this procedure. Despite this, the operation is not only invasive but also expensive, skill-based, and not generally accessible. Consequently, in clinical practice, non-invasive, repeatable methods that can replace HVPG would be crucial [20]. Furthermore, the diagnostic use of HVPG measurement may be limited, as it does not directly provide histological information regarding inflammation or fibrosis.

Percutaneous Fine-Needle Aspiration (FNA) and Core Biopsy for Focal Lesions

In patients with cirrhosis, localized liver lesions are evaluated with percutaneous FNA or core biopsy, especially to distinguish benign lesions from hepatocellular carcinoma (HCC). To acquire tissue samples for cytological or histological investigation, a tiny needle or core needle is inserted into the lesion under the guidance of ultrasonography or CT. Core biopsy enables the evaluation of tissue architecture and histological patterns, whereas FNA offers cytological examination. Although the method has high specificity for identifying cancers, there is a risk that tumors may develop along the needle tract, which occurs in 0.5-2% of cases. False-negative findings and sampling errors are also likely, especially in diverse lesions. Similar to liver biopsy complications, the biopsy site may experience bleeding, infection, and discomfort [21,22].

Endoscopic Ultrasound-Guided Liver Biopsy (EUS-LB)

Unlike percutaneous or transjugular approaches, EUS-LB uses real-time ultrasound imaging via an endoscope inserted into the gastrointestinal tract, enabling targeted sampling of both hepatic lobes, including deep-seated lesions or regions inaccessible to conventional methods. EUS-LB has gained prominence as a minimally invasive alternative to traditional liver biopsy techniques, offering distinct advantages in terms of safety, patient comfort, and diagnostic precision. This technique is especially beneficial for patients who require concurrent endoscopic evaluation (e.g., for assessing biliary obstruction or portal hypertension), as it combines multiple procedures into a single session, thereby reducing healthcare costs and patient inconvenience [23,24].

In terms of risks, EUS-LB has a low but significant rate of complications as follows: bleeding happens in 0-2.3% of cases, but it is usually minor and manageable endoscopically; rare complications (<1%) include infection, gastrointestinal perforation, or damage to adjacent organs during needle passage, which is lessened by real-time ultrasound guidance; and endoscopic procedures carry sedation-related risks, like respiratory depression, which necessitate careful patient selection, especially in frail patients [25,26]. Additionally, the cost and resource intensity prevent broad implementation, as EUS-LB necessitates costly sedative infrastructure and equipment (such as echoendoscopes and specialty needles), which makes it impractical in environments with limited resources.

Laparoscopic Liver Biopsy

Under general anesthesia, it is a minimally invasive surgical technique that uses a laparoscope to see and directly take samples of liver tissue through tiny abdominal incisions. Because it enables targeted sampling and simultaneous evaluation of abdominal pathology, such as portal hypertension or tumor staging, it is especially beneficial for patients with focal lesions (such as tumors or cysts) or those who are not candidates for percutaneous biopsy because of coagulopathy, ascites, or obesity [27]. Liver biopsy (LLB) is only available at tertiary care facilities due to its significant drawbacks, which include operator reliance and the need for specific surgical and laparoscopic ultrasound skills. Compared to percutaneous biopsies, specimen adequacy can vary, with fewer entire portal tracts, which may lower sensitivity for diffuse illnesses, such as cirrhosis, in their early stages [28]. Furthermore, the surgery is resource-intensive, requiring expensive operating room infrastructure and equipment (such as hemostatic devices and laparoscopes), which raises healthcare costs.

Non-invasive methods

Traditional Non-invasive Tools

The first-line imaging method for assessing liver cirrhosis is ultrasound due to its real-time imaging capabilities and safety. However, operator skill and subjective interpretation of qualitative characteristics, including liver surface nodularity, parenchymal heterogeneity, and portal vein Doppler flow patterns, have a significant impact on its diagnostic accuracy [29]. Its usefulness for early identification is limited since these characteristics frequently appear only in advanced stages of cirrhosis. According to research, early architectural alterations (such as minor nodularity) are frequently overlooked by conventional ultrasonography, which has a sensitivity of less than 70% for identifying severe fibrosis (F3-F4) and cirrhosis [4,29]. Although Doppler ultrasonography helps evaluate portal hypertension, it is unable to measure the course of liver fibrosis or hepatic stiffness, which is essential for early management [29].

CT scans offer high-resolution anatomical information, such as collateral circulation and liver morphology (such as splenomegaly or caudate lobe hypertrophy). However, CT's use in early diagnosis is limited, as these characteristics are typically only present in decompensated cirrhosis [29]. When ultrasonography was used to identify severe hepatic steatosis, the area under the curve (AUC) was 0.93 (95% confidence interval: 0.91-0.95). With AUCs of 0.761 and 0.807 for CT and ultrasonography, respectively, the detection of hepatic steatosis of at least 5% is significantly less accurate. These imaging techniques cannot detect early stages of fibrosis despite their ability to evaluate the nodular hepatic surface, caudate lobe hypertrophy, alterations in spleen size and vasculature, and morphologic abnormalities observed in cirrhosis [30].

Emerging Non-invasive Methods

Point-shear wave elastography (p-SWE), two-dimensional shear wave elastography (2D-SWE), and vibration-controlled transient elastography (VCTE, also known as FibroScan) are ultrasound elastography techniques. VCTE is a proven technique with good diagnostic and prognostic accuracy that is utilized globally [31].

Vibration-Controlled Transient Elastography (FibroScan)

Transient elastography (TE) is a non-invasive ultrasound technique that evaluates tissue, including liver stiffness, using shear wave velocity. Unlike surface waves, which move on the surface as their name suggests, they are the expression of elastic waves that move within an object's body. Shear waves are transverse, as opposed to longitudinal sound waves, meaning that the motion of the impacted tissue is perpendicular to the direction of wave propagation. As a result, the liver parenchyma quickly attenuates shear waves, which travel slowly (less than 50 m/s). The Langevin Institute developed the technique in 1995, and it was first used for food industry quality control. Since 2001, however, it has been used in medical practice under the name FibroScan (Paris, France: Echosens) [32].

Compared to liver biopsy, TE has the advantage of measuring a broader region of interest, namely a cylindrical liver segment that is 1 cm wide, 4 cm long, and 4.5 cm deep. Approximately 100 times the volume of the liver cylinder measured by liver biopsy is contained in this area, which has a capacity of 3 cm³ [32].

MR Elastography (MRE)

By visualizing the propagation of acoustic shear waves and using mathematical algorithms to create cross-sectional maps of the complicated shear modulus, magnetic resonance elastography (MRE), a sophisticated MRI-based technique, measures the stiffness of liver tissue. In patients with non-alcoholic fatty liver disease (NAFLD), this method has shown excellent diagnostic accuracy in identifying fibrosis, and it has also worked well in obese people [33]. Additionally, MRE is a non-invasive and reliable method for detecting advanced liver fibrosis in post-transplant patients; its diagnostic efficacy remains unaffected by inflammatory load, sex, or weight [34].

Serum Biomarkers

There are the following two types of serum markers: direct and indirect. The NAFLD Fibrosis Score (NFS) and the Fibrosis-4 (FIB-4) score are examples of indirect serum indicators that are derived using standard laboratory measures in conjunction with demographic information. Three serum biomarkers implicated in matrix turnover are measured via direct fibrogenesis-related serum biomarkers, such as the Enhanced Liver Fibrosis (ELF) test, which includes tissue inhibitor of metalloproteinase-1, hyaluronic acid, and N-terminal pro-collagen III peptide [35].

In identifying advanced fibrosis and cirrhosis in several chronic liver diseases (CLD), such as NAFLD, alcoholic liver disease (ALD), and viral hepatitis, the ELF test exhibits good to exceptional diagnostic performance. According to a study, ELF may be used as an alternative to liver biopsy in certain circumstances [36]. The sensitivity of the ELF test in ruling out fibrosis is high (>0.90). The study highlights that while biopsy remains the gold standard, physicians should modify the ELF criteria according to the prevalence of the condition [36]. When compared to FIB-4 and NAFLD Fibrosis Score (NFS), the ELF test demonstrated superior diagnostic accuracy for identifying advanced fibrosis when evaluated against transient elastography (TE). According to the study, ELF is useful for screening at-risk groups, as it reduces the number of unnecessary referrals compared to FIB-4 and NFS. Only individuals who tested positive for TE and ELF underwent a liver biopsy [37].

Advanced Imaging

Since multiparametric magnetic resonance imaging (mpMRI) enables the use of a wide range of sophisticated sequences that cover and analyze the complex spectrum of metabolic and cellular changes in the liver parenchyma, it is currently one of the most promising tools for assessing liver cirrhosis. As a result, some authors have speculated that mpMRI may eventually replace the need for a virtual liver biopsy. According to preliminary data, mpMRI offers a superior cost-benefit ratio than biopsy. Since the T1 relaxation time rises in tandem with an increase in extracellular fluid volume, which is indicative of fibrosis and inflammation, T1 mapping is an essential part of multiparametric procedures for liver evaluation [38].

With the availability of microbubble-based contrast agents, contrast-enhanced ultrasound (CEUS) has gained popularity as a minimally invasive procedure for assessing liver diseases. A recent meta-analysis of 12 studies, which included 844 patients with chronic liver disease, found that the sensitivity, specificity, favorable likelihood ratio, and negative likelihood ratio of the hepatic vein arrival time (HVAT) measured by CEUS for the detection of cirrhosis compared to liver biopsy were 0.83, 0.75, 3.45, and 0.28, respectively. Additionally, the summary diagnostic odds ratio (random-effects model) was 15.23, and the summary area under the receiver operating characteristic curve (AUROC) was 0.74, indicating increased diagnostic accuracy of the measurement of HVAT by CEUS to detect cirrhosis [39].

​Artificial Intelligence and Machine Learning

The diagnostic picture for liver cirrhosis has been significantly transformed by artificial intelligence (AI) and machine learning (ML), particularly due to advancements in image pattern recognition and serum biomarker-based prediction modeling.

Pattern Recognition in Imaging

AI-powered imaging methods have significantly enhanced the identification and categorization of liver diseases. Finding tiny patterns suggestive of liver disease has been made possible by radiomics, an AI technique that extracts quantitative characteristics from medical pictures. For example, deep learning algorithms, especially convolutional neural networks (CNNs), have demonstrated high accuracy in distinguishing between cirrhosis, fatty liver, and normal liver tissue using ultrasound images. According to research, 97.3% of these disorders could be correctly classified, highlighting the potential of AI in the diagnosis of liver illness [40,41].

Furthermore, by examining shear wave elastography (SWE) images, artificial intelligence (AI) models have been created to evaluate the stages of liver fibrosis in a study involving patients infected with the hepatitis B virus, a deep learning model trained on SWE images performed better in staging liver fibrosis than more conventional techniques, such as the fibrosis-4 (FIB-4) index and the aspartate aminotransferase to platelet ratio index (APRI). These developments reduce the need for liver biopsies by enabling precise, non-invasive assessments of liver fibrosis [40].

Predictive Modeling Using Serum Biomarkers

The prognostic accuracy for the development of liver disease has increased with the integration of AI with serum indicators. By utilizing machine learning algorithms to analyze plasma metabolomics data, the risk classification of cirrhosis patients has been improved. For instance, models with better discriminative abilities for cirrhosis risk prediction were produced by combining metabolomics data with conventional indices such as FIB-4 and APRI [42].

Additionally, artificial intelligence (AI) models have been developed to predict the effectiveness of therapies like lenvatinib in cases of incurable hepatocellular carcinoma (HCC). High predictive performance was demonstrated using a decision tree-based model that incorporated circulating angiogenic factors and clinical features, yielding an area under the curve (AUC) of 0.873. Furthermore, with an AUC of 0.832, machine-learning models have been developed to assess the risk of HCC in patients with cirrhosis associated with the hepatitis B virus who have low blood alpha-fetoprotein levels. These models improve patient outcomes by enabling tailored risk assessment and treatment planning [43,44]. The diagnosis and treatment of liver cirrhosis have been greatly enhanced by the integration of AI and ML in imaging and blood biomarker analysis. These technologies represent a significant improvement in hepatology by providing non-invasive, precise, and customized techniques.

Multimodal Diagnostic Approaches

The accuracy of diagnosing liver illness has dramatically increased with the integration of imaging techniques with biomarker analysis - multimodal diagnostic approaches. Clinicians can obtain a more comprehensive evaluation of liver health, particularly in conditions such as liver fibrosis and cirrhosis, by integrating multiple diagnostic techniques.

Combining Imaging and Biomarker Analysis

The cooperation of imaging techniques and biomarker evaluations enhances the identification and characterization of liver diseases. For example, individuals with severe fibrosis and non-alcoholic steatohepatitis (NASH) have been identified using multiparametric magnetic resonance imaging (MRI) in conjunction with blood biomarkers. Better patient classification and treatment were made possible by this composite approach's superior diagnostic performance over single-modality evaluations [45,46]. Contrast-enhanced ultrasound (CEUS) has also helped assess liver nodules in high-risk patients when it is integrated into a multimodality framework. This method enables real-time assessment of lesion vascularity, which enhances diagnostic confidence and complements findings from other imaging techniques [47].

Potential for Improved Diagnostic Accuracy

Imaging and biomarker data, when combined, overcome the drawbacks of each diagnostic technique alone. For instance, research has shown that the accuracy of liver fibrosis staging is significantly increased by combining grayscale ultrasound images with elastography through the use of transfer learning algorithms. This multimodal strategy demonstrated the promise of integrated diagnostics by outperforming models that used single imaging modalities [48].

Genetic and Molecular Markers: Circulating Cell-Free DNA and Epigenetic Modifications

A possible non-invasive biomarker for assessing liver disorders, such as cirrhosis and hepatocellular carcinoma (HCC), is circulating cell-free DNA (cfDNA). Apoptosis, necrosis, or other types of cell death release cfDNA into the circulation; its concentration, integrity, and pattern of fragmentation might reveal underlying hepatic disease processes. Numerous investigations have shown a correlation between the degree of hepatic fibrosis and the development of HCC and increased cfDNA levels and certain fragmentation patterns. For example, quantitative cfDNA investigations have shown that higher cfDNA concentrations are associated with more severe liver damage, which may facilitate early identification and tracking of disease progression [49].

The cfDNA epigenetic landscape provides an additional layer of diagnostic and prognostic information beyond quantitative alterations. cfDNA from individuals with liver cirrhosis and HCC has been found to have aberrant DNA methylation patterns, particularly in the promoter regions of genes implicated in cell cycle control, apoptosis, and fibrogenesis. The deregulation of gene expression that accompanies liver damage and malignant transformation is reflected in these epigenetic changes. For instance, cfDNA methylation profiling has been effectively utilized to identify early molecular changes prior to morphological abnormalities showing up in imaging investigations [49].

Furthermore, combining gene expression profiles - whether obtained from cell-free mRNA or circulating exosomal RNA - with cfDNA methylation data may further enhance diagnostic precision. These multifaceted methods allow for a more thorough evaluation of the molecular processes that underlie the development of liver disease. Combining these genetic and epigenetic indicators enables more individualized patient monitoring and may ultimately inform treatment decisions. The sensitivity and specificity of cfDNA and epigenetic tests are anticipated to increase with the development of high-throughput sequencing and digital polymerase chain reaction (PCR) technologies, therefore reinforcing their function in the early identification, prognostication, and treatment of liver cirrhosis and HCC [49].

Clinical validation and guidelines

Meta-Analyses of NIM vs. Biopsy

Forty studies and patients with chronic hepatitis B and C, alcohol, and other causes of cirrhosis were included in a major meta-analysis conducted by Tsochatzis et al. [50]. According to this research, TE's pooled sensitivity and specificity for identifying cirrhosis were 83% and 89%, respectively. Additionally, portal hypertension and other cirrhosis consequences can be predicted using transient elastography. TE demonstrated 90% sensitivity and 79% specificity in identifying severe portal hypertension in a comprehensive meta-analysis conducted by Shi et al. [51].

Using non-invasive tests, case-finding strategies for MASLD with liver fibrosis should be employed in individuals with abnormal liver enzymes, cardiometabolic risk factors, and/or radiological signs of hepatic steatosis, particularly if they also have type 2 diabetes (T2D) or obesity with another metabolic risk factor. A methodical strategy that employs successive imaging techniques (such as transient elastography) and blood-based scores (like FIB-4) is suitable for ruling out or advancing fibrosis, which is predictive of outcomes related to the liver [52]. Both the American Association for the Study of Liver Diseases (AASLD) and the European Association for the Study of the Liver (EASL) advocate the use of non-invasive imaging methods (NIMs) as effective tools for detecting liver fibrosis and monitoring disease progression [53,54]. Both organizations emphasize the importance of NIMs simultaneously.

AASLD Guidelines

Primary risk assessment: Non-invasive tests are increasingly utilized as an initial triage tool to identify individuals at risk of advanced fibrosis or cirrhosis, thereby reducing reliance on liver biopsy. These assessments facilitate earlier detection of clinically significant disease in asymptomatic or mildly symptomatic populations. Incorporating NITs into routine screening pathways improves workflow efficiency and allows broader patient access.

Referral for further evaluation: Abnormal findings from primary non-invasive assessments guide targeted referral to hepatology specialists for confirmatory diagnostic studies, imaging modalities, or advanced biomarker testing. This stepwise approach optimizes resource allocation and minimizes unnecessary specialist consultations. It also enhances diagnostic precision by integrating clinical, biochemical, and elastographic data.

Role in monitoring: Non-invasive methods play an essential role in longitudinal monitoring of fibrosis progression or regression in chronic liver disease. Serial measurements provide clinically meaningful trends that support treatment decisions, risk stratification, and surveillance planning. Their repeatability, safety profile, and cost-effectiveness make them suitable for ongoing disease management.

EASL recommends a liver stiffness measurement (LSM) of <8 kPa to rule out advanced fibrosis. If TE is unavailable, Enhanced Liver Fibrosis (ELF) <9.8, FibroMeter <0.45, FibroTest <0.48, or FIB-4 <1.3 can be used to rule out advanced fibrosis. LSM of ≥12-15 kPa provides high specificity for ruling in advanced fibrosis in ALD [55].

Advantages of non-invasive methods

Sensitivity and Specificity

Non-invasive techniques, especially transient elastography (TE), provide a high diagnostic accuracy for severe cirrhosis and fibrosis, according to meta-analyses. According to a thorough meta-analysis, for example, TE can identify substantial fibrosis with a sensitivity of around 80-85% and a specificity of 80-90%. When considering the sample errors associated with liver biopsy, these results are comparable to or even better than those obtained through liver biopsy. Other non-invasive blood indicators and grading systems have shown comparable outcomes, which support their application in clinical practice [56].

Reduced Risk and Complications

The non-invasive evaluation of cirrhosis and fibrosis aims to mitigate the risk of peri-procedural consequences, which is one of the limitations of liver biopsy. Overall, there is a strong correlation between biopsy scores and radiologic and serum indicators of fibrosis, particularly when cirrhosis (F4 or F<4) or fibrosis (F0 vs. F>0) is excluded. This characteristic frequently prevents liver biopsies [57].

Ease of Use and Accessibility

Liver fibrosis can be effectively and regularly monitored in the same patient, a significant benefit of non-invasive detection and evaluation of liver fibrosis. The benefits of serum biomarkers include strong repeatability and high applicability (>95%). Transient elastography and magnetic resonance elastography are the most promising methods because they yield accurate data for identifying advanced stages of fibrosis [58]. Most healthcare facilities offer a range of non-invasive techniques, including serum marker testing and ultrasound imaging. Performing specific non-invasive procedures, such as transient elastography, requires minimal training for medical personnel [58].

Accuracy and Reliability

When it comes to identifying advanced fibrosis, non-invasive imaging techniques and blood biomarkers have shown excellent diagnostic accuracy. Algorithms like APRI, the FIB-4 index, and NFS are simple to apply in a clinical environment since they make use of easily accessible patient demographics and laboratory data. The most validated non-invasive test (NIT), the FIB-4 index, should be used for primary risk assessment in patients at risk of MASLD development, according to the most recent AASLD recommendations [30].

Cost-Effectiveness

Non-invasive techniques are more cost-effective than liver biopsies, making them a more economical choice for diagnosing and monitoring liver disease. Non-invasive techniques can lessen the overall strain on healthcare resources by eliminating the necessity for liver biopsies.

Diagnostic Consistency

Liver biopsy has several drawbacks, such as interobserver uncertainty in histological interpretation and sampling variability. On the other hand, non-invasive techniques like serum biomarker panels and TE yield consistent and repeatable outcomes. Research has shown that remarkably similar results are obtained from repeated non-invasive assessments, ensuring accurate tracking of the disease's course over time.

Challenges and future directions

Limitations of Current Diagnostic Techniques

Serum biomarker panels, imaging-based modalities, and transient elastography are examples of non-invasive techniques that have demonstrated encouraging diagnostic results. However, the accuracy of transient elastography can be impacted by variables, including patient characteristics (such as obesity or ascites), which can result in varying sensitivity and specificity among various patient groups [59].

Need for Standardization of Non-invasive Methods

The lack of well-recognized methods and cutoff values is a significant obstacle to the widespread adoption of non-invasive diagnostic techniques. Reliability and reproducibility are ensured via standardization. Consensus recommendations that include pre-analytical factors (such as sample collection, handling, and processing), analytical techniques, and result interpretation should be the main focus of efforts [52]. Clinicians can gain more trust in the diagnostic and prognostic data provided by non-invasive techniques by aligning these parameters across various healthcare settings, which will ultimately facilitate their incorporation into standard practice.

Future Perspectives

Future studies should use sophisticated data analytics and machine learning algorithms to incorporate several diagnostic modalities, such as imaging data, serum biomarkers, and genetic/epigenetic profiles, in addition to resolving the present constraints and standardization concerns [60]. This combined strategy may enhance the precision of the diagnosis and provide a more comprehensive view of the course of liver disease. In the end, conquering these obstacles will be essential to integrating non-invasive diagnostic developments into standard clinical practice, increasing patient safety, cutting medical expenses, and boosting clinical management in general [61]. Here, we have summarized invasive vs. non-invasive diagnostic methods in Table 1.

**Table 1:** Comparison of invasive and non-invasive methods for liver cirrhosis assessment. HVPG: hepatic venous pressure gradient; EUS-LB: endoscopic ultrasound-guided liver biopsy; TE: transient elastography; MRE: magnetic resonance elastography; ELF: Enhanced Liver Fibrosis test; FIB-4: Fibrosis-4 index; AI: artificial intelligence; mpMRI: multiparametric magnetic resonance imaging; AASLD: American Association for the Study of Liver Diseases; EASL: European Association for the Study of the Liver; AUC: area under the curve FibroScan (Paris, France: Echosens)

Parameters	Invasive methods	Non-invasive methods
Examples	Percutaneous biopsy, HVPG, EUS-LB	TE (FibroScan), MRE, ELF, FIB-4, AI imaging, mpMRI
Accuracy (advanced fibrosis)	High (gold standard)	High (AUC >0.90 for TE, MRE)
Complications	Bleeding, infection, sampling error	Minimal to none
Repeatability	Limited	Easily repeatable
Patient acceptance	Low (pain, anxiety, sedation required)	High (non-invasive, outpatient-friendly)
Cost and accessibility	High cost, limited availability	Variable (some widely accessible, others need resources)
Use in special populations	Risky in coagulopathy, ascites	MRE/AI remains accurate in obesity, post-transplantation, and other conditions.
Guideline support	It is still considered a reference in complex cases	Strongly recommended (AASLD, EASL 2023-24)

## Conclusions

NIMs have emerged as transformative tools in the diagnosis and monitoring of liver cirrhosis, effectively addressing the limitations of traditional invasive approaches. As evidenced by growing clinical guidelines and technological advancements, techniques such as transient elastography, magnetic resonance elastography, serum biomarkers (e.g., ELF, FIB-4), and AI-driven imaging hold remarkable diagnostic accuracy, safety, and reproducibility. The integration of imaging, serological markers, and emerging genetic and epigenetic profiles, powered by artificial intelligence and machine learning, heralds a new era in precision hepatology. These multimodal diagnostic approaches not only enhance early detection but also enable dynamic disease monitoring, personalized risk assessment, and improved patient outcomes.

Nonetheless, challenges such as variability in diagnostic thresholds, cost, accessibility, and standardization across diverse populations remain. Future efforts must prioritize global accessibility, real-world validation, and the development of cost-effective, point-of-care diagnostics. Ultimately, NIMs are not just complementary tools - they are central to the future of liver disease management and may one day fully replace biopsy in routine clinical practice.
